# Supplementary material for: Comprehensive Annotation and Functional Exploration of MicroRNAs in Lettuce
Source: Front Plant Sci. 2021 Dec 24;12:781836. doi: 10.3389/fpls.2021.781836 (PMC8739914; doi:10.3389/fpls.2021.781836)
Supplement: Supplementary file 2 [file Data_Sheet_8.ZIP › GSM2230748.plot/Lsa-miR166a_Lsat_1_v5_gn_1_37081.1_1030_TPlot.pdf]

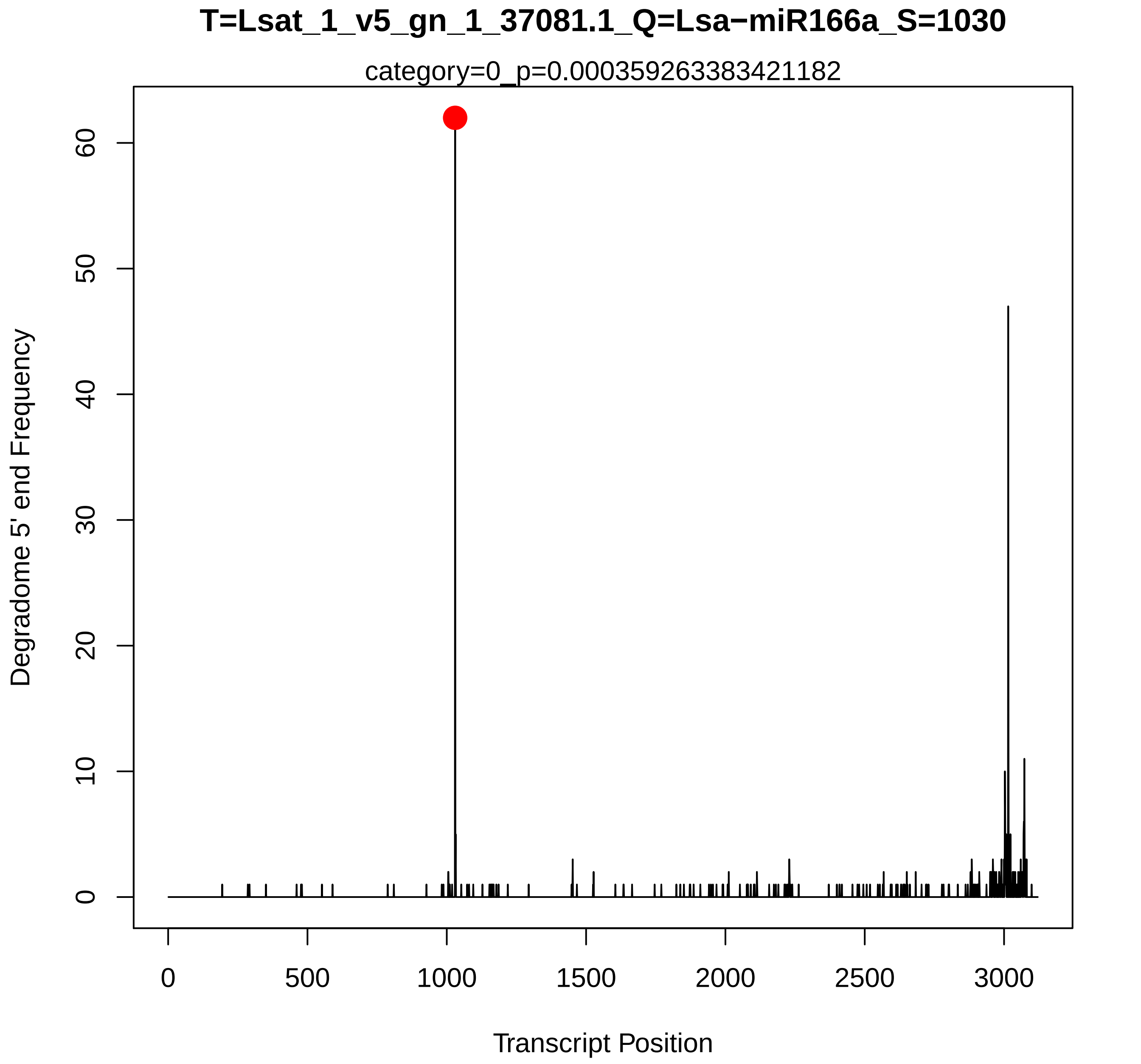

T=Lsat_1_v5_gn_1_37081.1_Q=Lsa−miR166a_S=1030
category=0_p=0.000359263383421182
Degradome 5' end Frequency
0
10
20
30
40
50
60
Transcript Position
0
500
1000
1500
2000
2500
3000
